# Genomic surveillance detects interregional spread of New Delhi metallo-beta-lactamase-1-producing *Providencia stuartii* in hospitals, Romania, December 2021 to September 2023

**DOI:** 10.2807/1560-7917.ES.2024.29.47.2400587

**Published:** 2024-11-21

**Authors:** Marius Linkevicius, Sandra Witteveen, Mariana Buzea, Mirela Flonta, Marina Indreas, Maria Nica, Edit Székely, Daniela Tălăpan, Olov Svartström, Erik Alm, Daniel Palm, Dominique L Monnet, Antoni PA Hendrickx, Anke Kohlenberg, Gabriel Adrian Popescu

**Affiliations:** 1 European Centre for Disease Prevention and Control, Stockholm, Sweden; 2 Centre for Infectious Disease Control (Cib), National Institute for Public Health and the Environment (RIVM), Bilthoven, The Netherlands; 3 Elias Hospital, Bucharest, Romania; 4 Spitalul Clinic de Boli Infecțioase, Cluj-Napoca, Romania; 5 Spitalul Judeţean de Urgenţă Bacău, Bacău, Romania; 6 Clinical Hospital of Infectious and Tropical Diseases “Dr. V. Babes” and “Carol Davila” UMF- Bucharest, Bucharest, Romania; 7 Targu Mures County Emergency Clinical Hospital, Targu Mures, Romania; 8 National Institute of Infectious Diseases “Prof. Dr. Matei Bals”, Bucharest, Romania; 9 Carol Davila University of Medicine and Pharmacy, Bucharest, Romania

**Keywords:** carbapenem-resistant Enterobacterales, *Providencia stuartii*, carbapenemase, NDM-1, surveillance, whole genome sequencing

## Abstract

**Background:**

New Delhi metallo-beta-lactamase (NDM)-producing *Providencia stuartii* has been reported from European Union/European Economic Area (EU/EEA) countries with increasing frequency. During 2018 to 2022, 355 cases of NDM-producing *P. stuartii* were detected in seven hospitals reporting on NDM-production in Enterobacterales in Romania.

**Aim:**

Our aim was to determine the extent of spread of NDM-producing *P. stuartii* in hospitals in Romania.

**Methods:**

We analysed whole genome sequences and epidemiological data of 74 *P. stuartii* isolates collected in six hospitals from December 2021 to September 2023.

**Results:**

We identified four multi-hospital clusters including isolates detected over more than a year, indicating sustained spread of *bla*_NDM-1_-carrying *P. stuartii* within the healthcare system. These clusters consisted of isolates from up to four hospitals and three regions. Three multi-hospital clusters were caused by a specific multidrug-resistant *P. stuartii* sequence type 46 lineage carrying *bla*_NDM-1_ and a large set of additional resistance markers. Investigation in an international context showed that this lineage had already been detected in nine countries (Bulgaria, France, Germany, Ireland, the Netherlands, Romania, Switzerland, United Kingdom, United States) since 2015.

**Conclusion:**

Our results alert about the risk of carbapenem-resistant *P. stuartii* transmission in healthcare settings. Enhanced infection prevention and control measures should be instituted as soon as cases are detected in healthcare facilities. National surveillance systems in EU/EEA countries should, in addition to carbapenem-resistant and/or carbapenemase-producing *Klebsiella pneumoniae* and *Escherichia coli*, consider reporting carbapenem-resistant and/or carbapenemase-producing *P. stuartii* and other Enterobacterales where relevant.

**Key public health message**

**What did you want to address in this study and why?**

Carbapenem-resistant Enterobacterales (CRE) cause difficult-to-treat infections in vulnerable hospitalised patients. A type of CRE so far very rare in Europe – NDM-1-producing *Providencia stuartii* – has been reported by hospitals in Romania. We conducted a genomic investigation to better understand how NDM-1-producing *P. stuartii* is spreading in hospitals and generate timely information for control.

**What have we learnt from this study?**

We identified four clusters of this pathogen involving several hospitals that extended over more than a year, indicating sustained transmission in healthcare facilities. Three of these clusters were caused by a specific *P. stuartii* sequence type 46 lineage. Transmission of this lineage within and between healthcare facilities had not been documented before, although it has already been detected in nine countries since 2015.

**What are the implications of your findings for public health?**

Sustained transmission in hospitals in Romania and the international spread point to high risk of further transmission of NDM-1-producing *P. stuartii* in healthcare settings. Enhanced infection prevention and control measures should be put in place as soon as cases are detected in healthcare facilities. National surveillance systems for CRE should include data collection, analysis and reporting for *P. stuartii* to monitor the extent of spread.

## Introduction

Molecular surveillance of carbapenemase-producing Enterobacterales (CPE) and related outbreak investigations in the European Union (EU)/European Economic Area (EEA) have so far mainly focused on *Klebsiella pneumoniae* and *Escherichia coli* [1,2]. However, in reply to an unpublished survey on the epidemiological situation of CPE conducted by the European Centre for Disease Prevention and Control (ECDC) in 2023, EU/EEA countries reported carbapenemase-producing isolates from several other genera and species of Enterobacterales. New Delhi metallo-beta-lactamase-1 (NDM-1)-producing *Providencia stuartii* received particular attention in various EU/EEA countries after its detection related to patient transfers from Ukraine [3].

While many EU/EEA countries detected few and mainly importation-related cases of NDM-producing *P. stuartii*, Romania reported 355 cases of NDM-producing *P. stuartii* over the period 2018 to 2022 from seven hospitals with the capacity to detect NDM-production in Enterobacterales (data not shown), pointing to potential spread within the country. Dissemination of NDM-1-producing *P. stuartii* in Romania had also been reported in a previous study based on isolates collected from January 2016 to September 2017 [4]. We piloted genomic surveillance with the aim to determine the extent of spread of NDM-producing *P. stuartii* in seven hospitals in the period 2021 to 2023 and generate timely information for national surveillance and control.

## Methods

### Sample selection

Hospitals that had detected isolates of carbapenem-resistant and/or carbapenemase-producing *P. stuartii* within 2 years before the start of the study were invited to participate. Each hospital was asked to submit up to 20 isolates of carbapenem-resistant and/or carbapenemase-producing *P. stuartii* with preference given to recent isolates from 2022 and 2023. Eligible for inclusion were* P. stuartii* isolates identified during clinical routine testing in hospital laboratories with resistance to any carbapenem (ertapenem, imipenem, meropenem) according to the European Committee on Antimicrobial Susceptibility Testing (EUCAST) breakpoints, or *P. stuartii* isolates confirmed as carrying carbapenemase genes. Therefore, isolates categorised as ‘susceptible’ (S) or ‘susceptible increased exposure’ (I) could have been included if confirmed as carbapenemase producers. Isolates from non-duplicate patients meeting the above definition were included retrospectively, with preference for isolates from clinical specimens (e.g. blood, urine, sputum, wound secretion).

### Epidemiological and microbiological data collection

Demographic and epidemiological data included age, sex (i.e. female, male, other or unknown), type of patient (i.e. inpatient or outpatient) and clinical significance (infection or colonisation/carriage status or unknown); in case of infection, we also included the site of infection. In addition, we collected the most likely mode of acquisition (i.e. healthcare- or community-associated, differentiated by a hospital stay of more or less than 48 h at the time of sample collection), an epidemiological link to another patient with *P. stuartii* infection or colonisation, previous travel within the past 12 months, previous hospitalisation within the past 12 months as well as direct hospital transfer from another country.

The collected microbiological data included a unique isolate identifier, the date of sample collection, a pseudonymised code of the healthcare institution submitting the sample, its National Territorial Unit for Statistics level 2 (NUTS 2) location, the type of sample, and routine antimicrobial susceptibility testing (AST) results for a panel of antibiotics including amoxicillin-clavulanic acid, piperacillin-tazobactam, cefotaxime, ceftazidime, cefepime, ceftazidime-avibactam, aztreonam, ertapenem, imipenem, meropenem, amikacin, gentamicin, tobramycin, ciprofloxacin, trimethoprim-sulfamethoxazole and fosfomycin. Isolates were tested with the routine methods currently in place at hospital laboratories. Disk diffusion zone diameters and minimum inhibitory concentrations were interpreted using EUCAST breakpoints v14.0 [5].

### Bioinformatic analysis

Assemblies were produced using SPAdes v3.15.5 and alleles were called with ChewBBACA v3.2.0 [6]. Clustering was performed with the core genome multilocus sequence typing (cgMLST) scheme developed by the National Institute for Public Health and the Environment (RIVM) comprising 3,079 core genes [3]. The cluster cut-off was set to 25 allelic differences (ADs). Sequence types (STs) were assigned using the Institute Pasteur-hosted *Providencia* spp. MLST scheme [7,8]. Antimicrobial resistance genes were identified using ResFinder v4.1.11 (database downloaded on 29 September 2022) with default settings [9]. Plasmid replicons were determined using PlasmidFinder v2.0.1 (database downloaded on 1 March 2023) [10,11]. An interactive phylogenetic tree based on neighbour-joining algorithm including relevant metadata was visualised in Microreact [12]. For comparison, we downloaded all *P. stuartii* genomes from National Center for Biotechnology Information (NCBI) on 22 April 2024.

The final dataset included (i) 74 *P. stuartii* genomes from hospitals in Romania collected for this study, (ii) 68 genomes from investigations in the EU/EEA including 60 published genomes [3], five unpublished *P. stuartii* genomes from Latvia linked to patient transfers from Ukraine and three *P. stuartii* genomes from an outbreak in Italy [13], and (iii) 236 other *P. stuartii* genomes from NCBI.

## Results

### Participation and geographical representativeness

**Figure 1:**
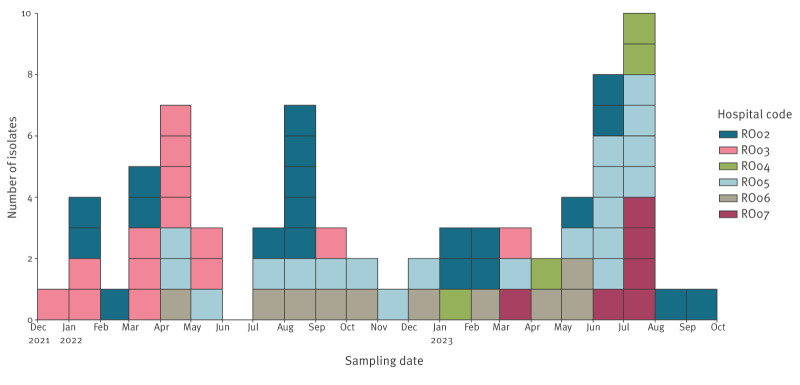
Time distribution of *Providencia stuartii* isolates by hospital, Romania, December 2021–September 2023 (n = 74)

Six of the seven initially registered hospitals submitted carbapenem-resistant and/or carbapenemase-producing *P. stuartii* isolates for whole genome sequencing. A total of 74 *P. stuartii* isolates were received from the hospitals: 20 from hospital RO02, 14 from hospital RO03, four from hospital RO04, 20 from hospital RO05, 10 from hospital RO06 and six from hospital RO07. The six participating hospitals were from the north, the centre and the capital of the country, and covered four of the eight NUTS 2 regions in Romania, i.e. București-Ilfov, Centru, Nord-Est and Nord-Vest. The first isolate was collected on 29 December 2021 and the last isolate on 5 September 2023 (Figure 1).

The time distribution should not be considered as an epidemic curve as it does not include all cases. We retrospectively selected only a subset of isolates (up to 20 per hospital). The variation over time may be affected by retrospective inclusion of stored *P. stuartii* isolates with a preference for the most recent isolates. The figure therefore does not reflect true temporal trends in incidence.

### Epidemiological and microbiological characteristics

Of 74 patients, more were male (n = 48) than female (n = 26), and the median age was 64.5 years (range: 19–91) based on complete data for these variables. Most (n = 72/74) isolates were reported as associated with an infection, in decreasing frequency: lower respiratory tract infection (n = 24/72), bloodstream or disseminated/systemic infection (n = 22/72), urinary tract infection (n = 18/72) and skin and soft tissue infection (n = 6/72). For two isolates, one collected from urine and one from an unspecified sample type, the site of infection was recorded as unknown. Fifty-eight of 74 isolates were categorised as healthcare-associated, while the mode of acquisition was unknown for the remaining isolates (n = 16/74). Many patients (n = 45/74) had had prior healthcare contact (direct hospital transfer or prior hospitalisation within 12 months), 24 of 74 patients did not have healthcare contact and for five patients, this information was unknown. No patient was reported to have travelled within 12 months before sampling, with travel history stated as no travel (n = 38/74) or unknown (n = 36/74). Information on prior residence in long-term care facilities was not available.

**Figure 2:**
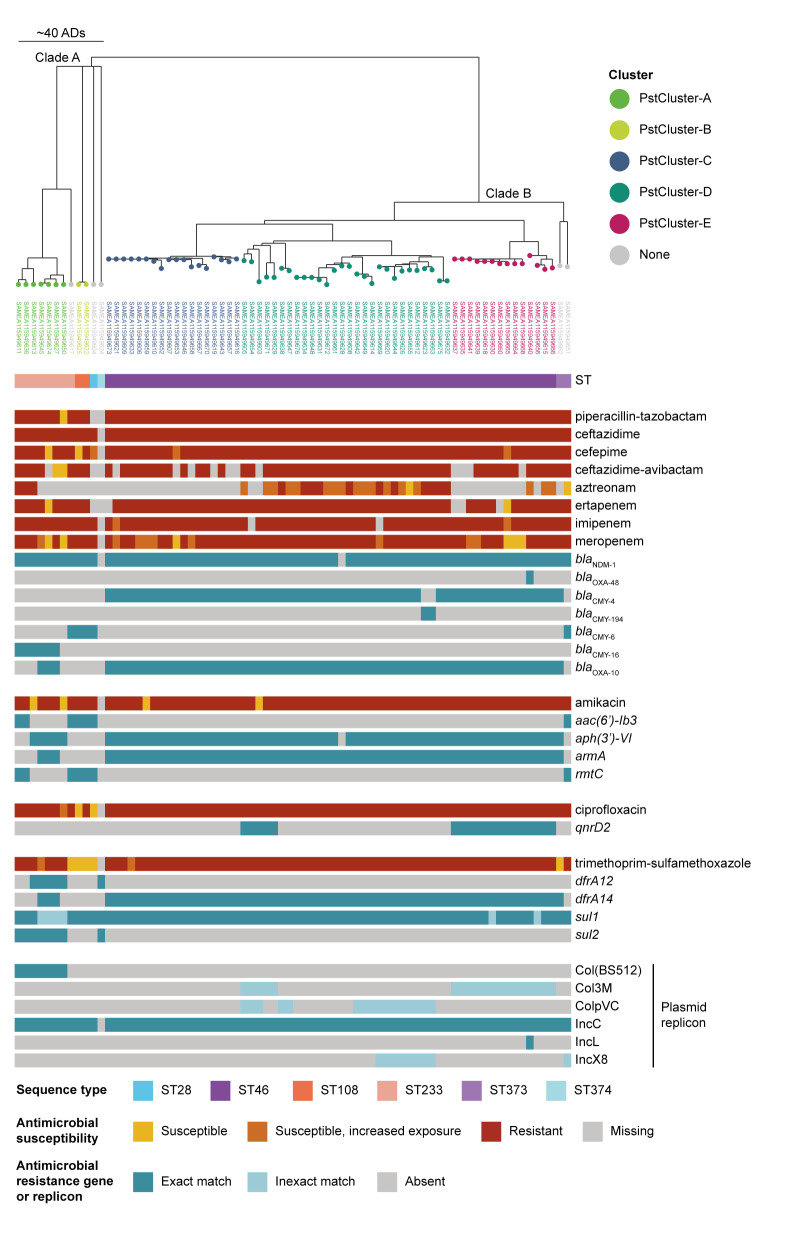
Phenotypic antimicrobial susceptibility testing results and relevant genotypic antimicrobial resistance results of *Providencia stuartii* isolates, by cluster, Romania, December 2021–September 2023 (n = 74)

AD: allelic difference; ST: sequence type.

As expected, based on their selection for carbapenemase production, most (> 90%) of the tested *P. stuartii* isolates were resistant to penicillins, cephalosporins and carbapenems, and therefore multidrug-resistant [14]. Most were also resistant to amikacin, ciprofloxacin and trimethoprim-sulfamethoxazole. Detailed results of routine AST for 72 of 74 isolates carrying *bla*_NDM-1_ are displayed in Table 1 and Figure 2. We do not report AST results for gentamicin and tobramycin due to intrinsic resistance of *P. stuartii* to these antibiotics [15], nor for fosfomycin due to the absence of valid clinical breakpoints, even though it is part of standard commercial AST panels for Enterobacterales [16].

**Table 1:** Routine antimicrobial susceptibility testing results for *Providencia stuartii* isolates carrying *bla*_NDM-1_, Romania, December 2021–September 2023 (n = 72)^a^

Antimicrobial	Tested isolates	Resistant isolates^b^	Percentage resistant
Piperacillin-tazobactam	71	70	99
Ceftazidime	72	72	100
Cefepime	72	67	93
Ceftazidime-avibactam	59	57	97
Aztreonam	32	14	44
Ertapenem	67	65	97
Imipenem	70	68	97
Meropenem	72	57	79
Amikacin	72	68	94
Ciprofloxacin	72	69	96
Trimethoprim-sulfamethoxazole	72	65	90

^a^ Two isolates in the dataset did not carry *bla*_NDM-1_ and are therefore excluded from this table.

^b ^Results were interpreted using European Committee on Antimicrobial Susceptibility Testing (EUCAST) clinical breakpoints v14.0 [5].

Results were interpreted using European Committee on Antimicrobial Susceptibility Testing (EUCAST) clinical breakpoints v14.0 [5]. Inexact match: < 100% coverage and/or < 100% identity.

### Genomic clusters and related resistance genes and plasmid replicons

**Figure 3:**
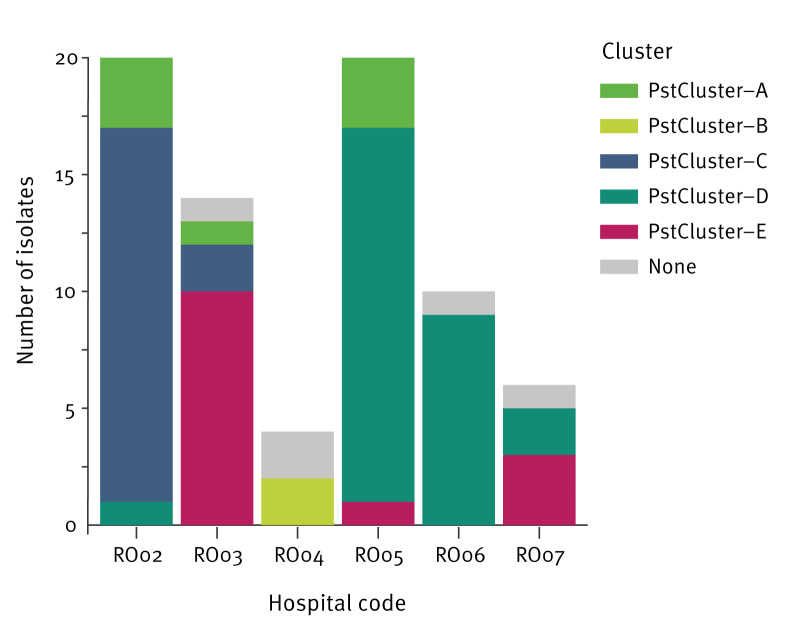
Presence of different clusters among *Providencia stuartii* isolates in six hospitals, Romania, December 2021–September 2023 (n = 74)

The phylogenetic tree of the *P. stuartii* isolates from Romania indicated a separation into two main clades: a smaller clade A with 12 isolates including ST28 (n = 1), ST108 (n = 2), ST233 (n = 8) and ST374 (n=1) as well as a larger clade B with 62 isolates of ST46 (n = 60) and ST373 (n = 2). We identified five clusters of potential recent transmission with a size ranging from two to 28 isolates (Table 2, Figure 2). Isolates of PstCluster-A (ST233) and PstCluster-B (ST108) belonged to the smaller clade A, while PstCluster-C, PstCluster-D and PstCluster-E (all ST46) were part of the larger clade B. All clusters except for PstCluster-B were multi-hospital clusters involving up to four hospitals and up to three NUTS 2 regions (Table 2). The three clusters with more than 10 isolates each (PstCluster-C, PstCluster-D and PstCluster-E) were located on the same branch within 44 ADs, indicating recent rapid expansion, while the two smaller clusters (PstCluster-A and PstCluster-B) were much more distant from each other and from PstCluster-C, PstCluster-D and PstCluster-E (Figure 2). All four multi-hospital clusters included isolates detected over more than a year confirming sustained transmission within the healthcare system (Table 2). In addition, *P. stuartii* isolates from up to three different clusters co-existed within the same hospital, indicating various separate transmission chains (Figure 3).

**Table 2:** Clusters of *Providencia stuartii* isolates, Romania, December 2021–September 2023 (n = 69 within-cluster isolates)

Clade	ST	PstCluster (25 ADs)	Number of involved hospitals	Number of isolates	Date of first isolate	Date of last isolate	NUTS 2 region(s)
A	233	A	3	7	22 Mar 2022	24 Jun 2023	București-Ilfov, Centru, Nord-Vest
	108	B	1	2	5 Jul 2023	10 Jul 2023	Nord-Est
B	46	C	2	18	2 Jan 2022	5 Sep 2023	Centru, Nord-Vest
		D	4	28	19 Apr 2022	31 Jul 2023	București-Ilfov, Nord-Vest
		E	3	14	29 Dec 2021	30 Jul 2023	București-Ilfov, Centru

AD: allelic difference; NUTS 2: National Territorial Unit for Statistics level 2; ST: sequence type.

Nearly all within-cluster isolates, i.e. 68 of 69 isolates, carried the metallo-beta-lactamase (MBL) gene *bla*_NDM‑1_, while the remainder of the resistome differed between the clades despite a similar AST profile (Figure 2). Of the 68 *bla*_NDM‑1_-positive within-cluster isolates, most (n = 62) co-carried two additional beta-lactamase genes, i.e. *bla*_OXA‑10_ and one *bla*_CMY_ gene variant (*bla*_CMY‑4_ or *bla*_CMY‑16_). Of the isolates with *bla*_CMY‑4_, two had inexact *bla*_CMY‑4_ matches and carried the same nucleotide mutation T757A resulting in a W253R amino acid change. These genes have been designated as *bla*_CMY‑194_. Five isolates had only one additional beta-lactamase gene, either *bla*_CMY‑6_ or *bla*_CMY‑16_, and one isolate did not carry any additional beta-lactamase gene. The plasmid-mediated AmpC beta-lactamase gene variants differed between clades and clusters, with *bla*_CMY‑16_ detected in six of seven isolates of PstCluster-A, *bla*_CMY‑6_ found in both isolates of PstCluster-B, and *bla*_CMY‑4_ found in isolates from PstCluster-C, PstCluster-D and PstCluster-E. Two PstCluster-D isolates carried *bla*_CMY‑194_. In addition to *bla*_NDM‑1_, *bla*_CMY‑4_ and *bla*_OXA‑10_, one isolate in PstCluster-E also carried the *bla*_OXA‑48_ carbapenemase gene (Figure 2).

Although detection of *bla*_NDM‑1_ alone was associated with resistance to most of the beta-lactam antibiotics included in Figure 2 except aztreonam, nine within-cluster isolates remained susceptible to various beta-lactams. Eleven of 14 aztreonam-resistant within-cluster isolates carried a combination of three beta-lactamase genes, i.e. *bla*_NDM‑1_, *bla*_CMY‑4_/*bla*_CMY‑194_ and *bla*_OXA‑10_, while the remaining three isolates carried *bla*_NDM-1_ and *bla*_CMY‑16_. Seventeen aztreonam-‘susceptible, increased exposure’ (I) isolates and one aztreonam-‘susceptible’ (S) isolate also carried the same above-mentioned triple combination of beta-lactamase genes.

For amikacin, all within-cluster isolates were predicted to be resistant by carrying the combination of two aminoglycoside resistance genes, either *armA* and *aph(3’)-VI* (n = 62) or *aac(6’)-Ib3* and *rmtC* (n = 4), or single genes, i.e. *aph(3’)-VI* (n = 2) or *armA* (n = 1). Despite the presence of these genes, four isolates (two in each clade) were susceptible to amikacin according to phenotypic results. Regarding quinolone resistance, all isolates in PstCluster-E (n = 14) and five PstCluster-D isolates carried the *qnrD2* gene encoding resistance to ciprofloxacin. The remaining 50 within-cluster isolates did not carry any acquired fluoroquinolone resistance genes, suggesting that chromosomal mutations may be the main explanation for the high proportion (67/69 within-cluster isolates) of ciprofloxacin resistance.

Trimethoprim resistance was predicted by carriage of the *dfrA14* gene by all isolates within PstCluster-C, PstCluster-D and PstCluster-E (n = 60) and three isolates of PstCluster-A, as well as the *dfrA12* gene only detected in five isolates from PstCluster-A. Isolates in PstCluster-B did not carry any detected trimethoprim resistance genes. The sulfamethoxazole resistance gene *sul1* was found in all within-cluster isolates (including six inexact matches) followed by the *sul2* gene only detected in isolates from PstCluster-A. Phenotypic trimethoprim-sulfamethoxazole resistance was confirmed for most (65/69) within-cluster isolates.

Various plasmid replicons were present in different clusters (Figure 2). All within-cluster isolates (n = 69) carried an IncC replicon, while other replicons were only present in the specific clusters. The Col(BS512) replicon was detected only in PstCluster-A isolates (seven of seven isolates), the ColpVC and IncX8 replicons only in PstCluster-D isolates (16 and eight of 28 isolates, respectively), and one isolate in PstCluster-E carried an IncL replicon. Two clusters (PstCluster-D: five of 28 isolates; PstCluster-E: 14 of 14 isolates) harboured a Col3M replicon.

### International context

**Figure 4:**
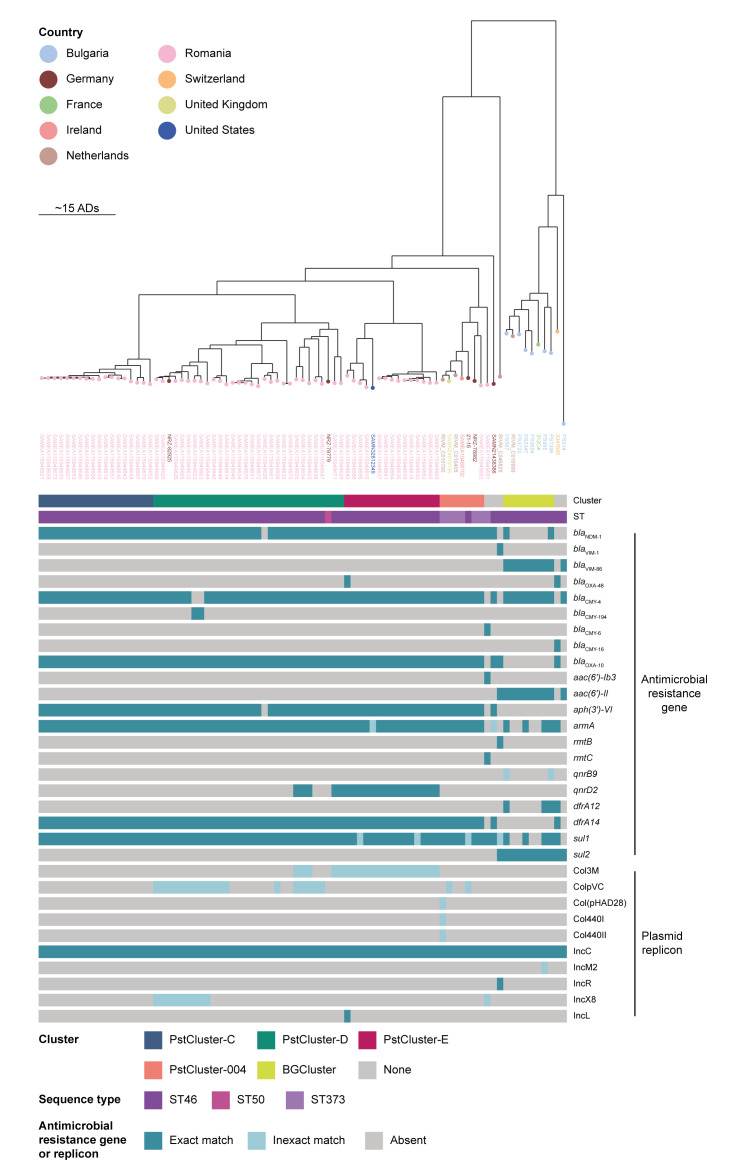
Phylogenetic tree of *Providencia stuartii* isolates belonging to the ST46 lineage and carrying carbapenemase genes, Romania and other countries, 2015–2023 (n = 83 isolates)

AD: allelic difference; ST: sequence type.

Addition of data from the public domain showed that two isolates from Germany clustered within PstCluster-D (NRZ-82925 and NRZ-79779) and one isolate from the United States (US) (BioSample accession number: SAMN32812349) within PstCluster-E with similar resistomes (Figure 4). Moreover, PstCluster-C, PstCluster-D and PstCluster-E had a distance of 27, 21 and 21 ADs, respectively, from PstCluster-004 consisting of two isolates from Germany and two isolates from the Netherlands from the European investigation into spread of *P. stuartii* related to medical transfers from Ukraine [3] (Figure 4). In addition, one isolate from Ireland (SAMEA10468702), one isolate from the United Kingdom (UK) (SAMN24019195) and one isolate from Romania (SAMEA115949662) clustered within PstCluster-004. For one German and one Dutch isolate from this cluster, there was related information on hospitalisation of the respective patients in Hungary [3]. All these isolates carried *bla*_NDM‑1_, *bla*_CMY‑4_, *bla*_OXA‑10_, *aph(3’)-VI*, *armA*, *dfrA14* and *sul1* resistance genes, known to confer resistance to clinically relevant antibiotics, and an IncC replicon (Figure 4).

Inexact match: < 100% coverage and/or < 100% identity.

On a neighbouring branch of the clade with PstCluster-C, PstCluster-D and PstCluster-E as well as PstCluster-004, we identified a clade containing seven isolates from Bulgaria and one isolate each from France (312C4), the Netherlands (RIVM_C016999) and Switzerland (3347685), the latter from a patient transferred from a hospital in North Macedonia [17], with the distance of ca 80 ADs in the phylogenetic tree (Figure 4). Within this clade, six isolates originating from three different hospitals in Bulgaria and isolates from France and the Netherlands formed a multi-country, multi-hospital cluster (BGCluster) of VIM-86-producing *P. stuartii* [18,19], also harbouring *bla*_CMY-4_, *aac(6’)-Il* and *sul2* as well as an IncC plasmid (Figure 4). Some of the cluster isolates also carried other relevant resistance genes including *bla*_NDM‑1_, *armA*, *qnrB9*, *dfrA12* and *sul1* and an IncM2 replicon (Figure 4).

## Discussion

We report the results of an epidemiological and genomic investigation in six hospitals in Romania performed to determine the extent of spread of NDM-producing *P. stuartii*. Among the 74 isolates submitted for this study, we found four multi-hospital clusters of *P. stuartii* carrying *bla*_NDM-1_, including isolates that were detected over more than a year pointing to sustained spread of NDM-producing *P. stuartii* within the healthcare system in Romania. Clusters included isolates from up to four hospitals and three different regions, indicating interregional spread. The phylogenetic tree revealed that several clusters were part of distinct clades, suggesting that NDM-1-producing *P. stuartii* has already been circulating and diversifying in the healthcare system over a longer time. This is confirmed by a previous study of NDM-1-producing *P. stuartii* in Romania in 2016 and 2017 [4] as well as the detection of an NDM-1-producing *P. stuartii* isolate in a patient transferred from Romania to the Netherlands in 2015 [20].

While outbreaks of NDM-1-producing *P. stuartii* have been detected previously [13,21], this study documents sustained healthcare-associated transmission and various transmission chains co-existing in hospitals based on whole genome sequencing results. Various characteristics of the MBL gene-carrying *P. stuartii* ST46 lineage, including its single locus variants ST50 and ST373, point to a similar behaviour as described for high-risk clones of carbapenemase-producing *K. pneumoniae* [22]. This lineage has already spread to nine countries, i.e. Bulgaria, France, Germany, Ireland, the Netherlands, Romania, Switzerland, the UK and the US, as documented by our analysis. Isolates of the *P. stuartii* ST46 lineage also carry a large set of resistance determinants. The lineage shows sustained healthcare-associated and interregional transmission in Romania for more than a year and has the capacity to cause infections at various sites as documented in this study.

All isolates of the ST46 lineage carried an IncC plasmid replicon, which has been described as the main plasmid replicon carrying MBL genes in *P. stuartii* isolates in numerous studies [3,4,13,17-19,21,23]. Several studies also demonstrated the presence of the *bla*_NDM-1_ gene on the IncC plasmid that could be transferred to other Enterobacterales, both in vivo [3] and in vitro [3,18,24]. Even though long-read sequences were not available in this investigation to definitively determine the location of the *bla*_NDM‑1_ gene, nearly 95% of *bla*_NDM‑1_-carrying isolates in the whole dataset contained an IncC replicon, indicating that this gene might reside on an IncC plasmid backbone.

Isolates of NDM-producing *P. stuartii* have been detected worldwide [23,25-29]. In the EU/EEA, NDM-producing *P. stuartii* mainly received attention after detection related to patient transfers from Ukraine [3]. However, there is evidence for a wider dissemination in Eastern Europe and the Balkan region including Bulgaria [18], Greece [24], Hungary (described epidemiological links) [3], North Macedonia (described epidemiological link) [17], Romania (this study) and Serbia (described epidemiological link) [30]. National surveillance data on carbapenemase-producing *P. stuartii* in EU/EEA and EU enlargement countries is urgently needed to delineate the extent of its spread and to guide control measures. In addition, research into duration of carriage, pathogenicity and population structure of *P. stuartii* would be useful to further define its capacity to develop and sustain healthcare-associated high-risk clones.

This investigation has several limitations. The selected hospitals do not cover all NUTS 2 regions of Romania. Isolates were included retrospectively, and inclusion was restricted to a maximum of 20 isolates by hospital. Evidence on the extent of asymptomatic carriage of NDM-producing *P. stuartii* is very limited. However, systematic screening for carriage in analogy to screening for other carbapenem-resistant Enterobacterales species was not performed in this study. The extent of spread of NDM-producing *P. stuartii* may therefore have been underestimated. In addition, AST for the potentially effective newer substances such as cefiderocol and aztreonam-avibactam was not performed and clinical information on antimicrobial treatment and respective outcomes was not collected.

## Conclusion

Public health professionals, clinicians, clinical microbiologists, and infection prevention and control practitioners should be vigilant about the risk of *P. stuartii* transmission in healthcare settings. Enhanced infection prevention and control measures in line with national and international guidance should be instituted as soon as cases are detected in healthcare facilities. National surveillance systems in EU/EEA countries should, in addition to carbapenem-resistant and/or carbapenemase-producing *K. pneumoniae* and *E. coli*, consider inclusion of data collection, analysis and reporting of carbapenem-resistant and/or carbapenemase-producing *P. stuartii* and other Enterobacterales where relevant.
